# Canine retraction and anchorage loss self-ligating versus conventional brackets: a systematic review and meta-analysis

**DOI:** 10.1186/s12903-015-0127-2

**Published:** 2015-11-04

**Authors:** Qiaozhen Zhou, Abdul Azeem Amin ul Haq, Liu Tian, Xiaofeng Chen, Kui Huang, Yu Zhou

**Affiliations:** Department of Prosthodontics, School and Hospital of Stomatology, Wenzhou Medical University, Wenzhou, China; International Education College, Wenzhou Medical University, Wenzhou, China; Department of Stomatology, Ningbo NO.2 Hospital, Ningbo, PR China; Department of Orthodontics, Hospital of Stomatology, Wenzhou Medical University, Wenzhou, 113 West College Road, 325000 wenzhou, China

**Keywords:** Self-ligating bracket, Orthodontic treatment, Systematic review, Anchorage loss

## Abstract

**Background:**

The purpose of this systematic review is to identify and review the orthodontic literature with regards to assessing possible differences in canine retraction rate and the amount of antero-posterior anchorage (AP) loss during maxillary canine retraction, using conventional brackets (CBs) and self-ligating brackets (SLBs).

**Methods:**

An electronic search without time or language restrictions was undertake in September 2014 in the following electronic databases: The Cochrane Oral Health Group’s Trials Register, CENTRAL, MEDLINE via OVID, EMBASE via OVID, Web of science. We also searched the reference lists of relevant articles. Quality assessment of the included articles was performed. Two of the authors were responsible for study selection, validity assessment and data extraction.

**Results:**

Six studies met the inclusion criteria, including 2 randomized controlled trials and 4 control clinical studies. One was assessed as being at low risk of bias. Five trials were assessed as being at moderate risk of bias. The meta-analysis from 6 eligible studies showed that no statistically significant difference was observed between the 2 groups in the rate of canine retraction and loss of antero-posterior anchorage of the molars.

**Conclusion:**

There is some evidence from this review that both brackets showed the same rate of canine retraction and loss of antero-posterior anchorage of the molars. The results of the present systematic review should be viewed with caution due to the presence of uncontrolled interpreted factors in the included studies. Further well-designed and conducted randomized controlled trials are required, to facilitate comparisons of the results.

## Background

Friction in sliding mechanics has drawn a lot of attention, especially as it pertains to effectiveness and efficiency in orthodontic tooth movement. The claim of reduced friction with self-ligating brackets (SLBs) is often cited as a primary advantage over conventional brackets(CBs). It would be logical, therefore, to assume that spaces could be closed faster since it is known that friction could influence movement rates and molar anchorage loss is also reduced as a result of the smaller load on the anchor unit. Although this concept is conceivable, clinical evidence is lacking to support the claim as the vast majority of the literature with contradictory findings. Recent systematic reviews also failed to report the superiority of SLBs over CBs when tooth movement rate was assessed [1, 2], challenging what logically would make sense.

Canine retraction is probably the most common clinical situation where sliding mechanics are used to move a tooth over a relatively long distance.

Therefore, it would be interesting to evaluate the “superiority” of one bracket over the other regarding friction, but to date only three studies have compared the rate of canine retraction using SLBs and CBs [3–5],and their results are controversial. Two [3, 4] have failed to find differences between those brackets, while the remaining one favored CBs. Even though the design of the latter study [5] allowed a more complete evaluation since full canine retraction was evaluated, measurements were taken directly in the mouth and rounded to the half millimeter, which could explain the reason of small differences found. Additionally, no information on tipping was collected, since differences in tipping could explain the differences found.

Another claim regarding SLBs, involves the belief that they would allow less AP anchorage loss of the molars during space closure. This idea comes from the theory that less friction would allow lighter forces to retract anterior teeth and, therefore, suboptimal forces would be applied to the posterior teeth [6]. Three clinical trials have examined this hypothesis [3, 7, 8], but only one evaluated the loss of AP anchorage during canine retraction and the follow-up was only over a period of 12 weeks. Three months may not be enough to detect differences between brackets, a longer period of evaluation could be more desirable.

Thus, the aim of this systematic review was to assess possible differences in canine retraction rate and the amount of AP anchorage loss during maxillary canine retraction, using CBs and SBs.

## Methods

This systematic review and meta-analysis was conducted according to the guidelines of the Preferred Reporting Items for Systematic reviews and Meta-Analyses (PRISMA) statement and the Cochrane Handbook. A review protocol does not exist.

### Study selection criteria

To be included in the review, trials had to meet the following selection criteria:

- Study design: Randomized or controlled clinical trials.
- Participants: Patients with full arch, fixed orthodontic appliance(s) treated with SLBs or (CBs).
- Interventions: Fixed appliance orthodontic treatment involving SLBs or CBs.
- Outcome measures: The outcome measures were canine retraction rate and the amount of AP anchorage loss related to both SLB and CB systems.

The exclusion criteria were (1) animal studies; (2) studies with no comparison group; and (3) editorials, opinions, or philosophy articles with no subjects or analytical design.(4)studies that used TADs in them.

### Search methods for identification of studies

An electronic search without time or language restrictions was undertaken in September 2014 in the following electronic databases: The Cochrane Oral Health Group’s Trials Register, CENTRAL, MEDLINE via OVID, EMBASE via OVID, Web of science. For the identification of studies included or considered for this review, detailed search strategies were developed for each database searched. These were based on the search strategy developed for MEDLINE (OVID) but revised appropriately for each database, see Table 1.

**Table 1:** Search strategy

1.	exp self-ligating bracket
2.	“self-ligating bracket”.mp.
3.	self-ligating bracket or self-ligate bracket.mp.
4.	“canine retraction”.mp.
5.	(canine retraction adj3 canine retraction velocity) or (conventional acid etching adj3 adhensive) or (molar anchorage loss adj3 anchorage loss).mp.
6.	Or/1-5

The above subject search was linked to the Cochrane Highly Sensitive Search Strategy (CHSSS) for identifying randomised trials in MEDLINE: sensitivity maximising version (2008 revision) as referenced in Chapter 6.4.11.1 and detailed in box 6.4.c of the Cochrane Handbook for Systematic Reviews of Interventions, Version 5.1.0 [updated March 2011]

1. randomized controlled trial.pt

2. controlled clinical trial.pt

3. randomized.ab

4. placebo.ab

5. drug therapy.fs

6. randomly.ab

7. trial.ab

8. groups.ab

9. or/1-8

10. exp animals/not humans.sh

11. 9 not 10

A manual search of orthodontic journals including American Journal of Orthodontics and Dentofacial Orthopedics, European Journal of Orthodontics, Angle Orthodontist, Journal of Orthodontics, and World Journal of Orthodontics were also performed.

We checked the bibliographies of the included papers and relevant review articles for studies not identified by the search strategies mentioned above. We contacted the authors of published papers and included studies to identify unpublished or ongoing trials.

### Selection of studies

At least two review authors independently scanned the list of titles and abstracts of potentially eligible studies. For studies appearing to meet the inclusion criteria, for which there were insufficient data in the title and/or the abstract to make a clear decision, the full paper was obtained. Any disagreement would be resolved by discussions with a third investigator.

### Quality assessment

For randomized controlled trials, 5 criteria were used for assessment: (1) randomization described, (2) allocation concealment reported, (3) intention-to-treat analysis performed, (4) blind assessment stated, and (5) a prior power calculation performed.

For cohort and cross-sectional studies, these criteria were used: (1) representative sample of adequate size, (2) well-matched samples, (3) adjustment for confounder in analyses, (4) blinded assessment stated, and (5) dropouts reported (for prospective studies only).

One point was given to each criterion if fulfilled. Half a point was granted if part of the criterion was met. Studies with less than 2 points were considered to be at high risk for bias; from 2 to less than 4 points the risk for bias was considered moderate; and for 4 points and above, the risk of bias was considered low. In areas of disagreement, a third investigator was consulted, and consensus was achieved after discussion.

All quality ratings have limitations, and our intention was to provide a relative scale to judge the quality of the chosen studies, by using the parameters stated above.

### Data extraction and analysis

At least two review authors assessed all included studies, to confirm eligibility, assess risk of bias and extract data. The following data was extracted: study designs, participants, interventions, and outcome measurements.

Meta-analyses would also be possible only on studies reporting the same outcomes at similar time intervals. A meta-analysis was performed to combine comparable results in each category by using Review Manager (version 5.2.11, The Nordic Cochrane Centre, The Cochrane Collaboration, Copenhagen, Denmark, 2014). Heterogeneity was assessed among the included studies. Results with less heterogeneity (I^2^ statistics < 75 %) were presented with a fixed- effect model, whereas results with I^2^ > 75 % utilized a random-effect model. Weighted mean differences were used to construct forest plots of continuous data. Odds ratios were used for dichotomous data. If there were a sufficient number of trials (more than 10) included in any meta-analysis, publication bias was to be assessed according to the recommendations on testing for funnel plot asymmetry as described in the Cochrane Handbook.

## Results

### Study selection and description of studies

**Fig. 1:**
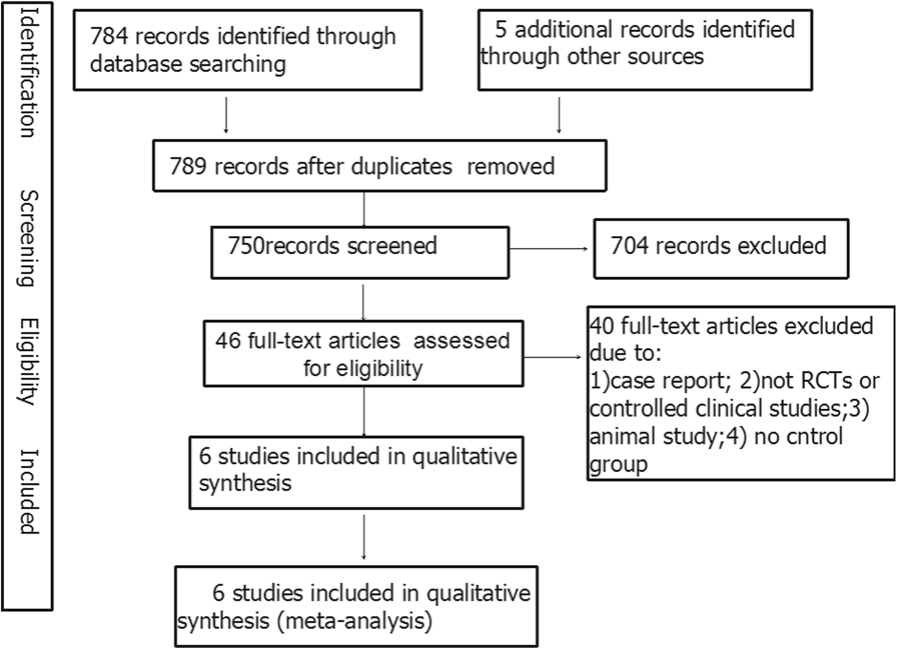
Flow figure

The agreement between the two independent review authors with regarding to article screening was almost perfect (kappa = 0.922). The flow diagram (See Fig. 1) describes the results of search queries. We initially identified a total of 789 references and 46 reports of trials as eligible according to the defined inclusion criteria for this review. The full-text of the remaining 34 articles led to the exclusion of 12 because they did not meet the inclusion criteria(6 were case reports, 7 were animal studies, 10 were not including control group, 7 were lab studies, and 4 were not RCT or control clinical studies). Additional hand-searching of the reference lists of selected studies did not yield additional papers. Thus, a total of 6 publications are included in the review. The details of each studies are presented in Table 2.

**Table 2:** Summarized data of the 6 included studies

Authors, year	Study design	Participants size, gender, age	Intervention, end point	Outcome measure	Outcome and authors conclusions	Notes
de Almeida [8]	retrospective cohort design	Group 1:23 patients (18 females and 5 males) with an initial mean age of 15.36 years (SD =5.59 years)	Group 1: 23 patients, with preadjusted conventional brackets (CBs).	Maxillary molar anchorage loss; the incisor tip	There were no significant differences in the amount of anchorage loss of the maxillary first molars and incisor tip change between SLB and CB systems during space closure. group.	Canines were retracted separately by means of NiTi coil springs (150 g of force) from the first molars
		Group 2: 15 patients (10 females and 5 males) with an initial mean age of 17.63 years (SD = 8.93 years)	Group 2: 15 patients with self-ligating brackets (SLBs).			
			End point: premolar space was closed			
Oz 2012 [4]	Prospective split-mouth design	19 orthodontic patients (5 male, 14 female) with a mean age of 13.6 years (range, 12.7 to 15.3 years)	The canine was bonded with an SC bracket on one side and MT brackets ligated with stainlesssteel ligature wires on the other side.	distal canine movement; The angular movement of the canines and molars was also evaluated	It is suggested that the rate of canine distalization was not different between the two groups	The mini-implants that were used in this study
			End point: 8 weeks after the start of canine distalization			
Machibya [7]	retrospective cohort	The study included 69 completed cases with mean age of 15.64 6 3.74 years at the start of treatment.	The first group (SLB) consisted of 34 patients treated by SmartClip (3 M Unitek, Monrovia, Calif) brackets. The second group (CB) consisted of 35 patients treated by conventional preadjusted Victory series brackets (3 M Unitek) tightly ligated with SS 0.020-inch ligatures.	Maxillary and mandibular molar anchorage loss; Incisor tipping	There were no significant differences in the amount of anchorage loss of the maxillary first molars and incisor tip change between SLB and CB systems during space closure. group.	The teeth were retracted down a 0.018-inch stainless steel archwire, using a medium Sentalloy retraction spring (150 g).
			End point: premolar space was closed			
Burrow [5]	Prospective split-mouth design	A sample size of 43 patients (14.8 + 6.24 year,44 % Female 56 % Male) was used in this investigation (21 Damon3, 22 SmartClip, 43 conventional Victory Series).	Each patient had a 0.022-inch slot conventional bracket placed on one canine and a 0.022-inch slot Damon3 or SmartClip bracket placed on the other, with the left or right side for the self-ligating bracket chosen using a randomization sequence.	Rate of Movement	The retraction rate is faster with the conventional bracket, probably because of the narrower bracket width of the self-ligating brackets.	transpalatal arch was placed
			End point:one of the canines was in the proper position			The canines were retracted using a GAC Sentalloy retraction spring (150 g).
Mezomo [3]	RCT	The sample comprised 15 healthy patients (10 girls and five boys), between the ages of 12 and 26 years (mean, 18 years	In a random, split-mouth design, self-ligating brackets (SmartClip, 3 M-Unitek) and conventional brackets (Gemini, 3 M-Unitek) were bonded to the right and left sides of all patients by raffle.	Average Rates of Distal Movement of Upper Canines	Distal movement of the upper canines and anchorage loss of the first molars were similar with both conventional and self-ligating brackets. Rotation of the upper canines during sliding mechanics was minimized with self-ligating brackets.	
			END POINT:12 weeks (T3) of canine retraction	Total Rotation of Canines		
				Anchorage Loss of Upper First Molars		
				Average Rates of Distal Movement of Upper Canines		
Monini [9]	RCT	The sample comprised 25 healthy patients	Through block randomization, one maxillary canine was bonded with a 0.022-inch SLB (In-Ovation R, GAC), while the other received a 0.022-inch CB (Ovation, GAC).	Canine and Molar Inclinations	Both brackets showed the same velocity of canine retraction and loss of anteroposterior anchorage of the molars. No changes were found between brackets regarding the inclination of canines and first molars.	No auxiliary devices such as transpalatal arches, headgear, or elastics were used. Nickel-titanium closed coil springs (CCS) of 100 g (GAC) were activated for 17 mm and secured from the hooks of first molars to the hooks of the canine brackets with ligature wires.
			End point:after total canine retraction	Time taken for total space closure		
				Canine Retraction Velocity		

### Risk of bias

The methodological quality of the trials considered in the review is presented in Table 3. Of the 6 studies, 1 was judged to have a low risk of bias, 5 were categorized as having moderate risk (Table 3). The 1 study with low risk of bias was the randomized controlled trial. The other cohort studies were judged to have moderate risk of bias. The most recurrent shortcomings were blind assessments reporting drop-outs and allocation concealment with no methods of sequence generation described. Furthermore, only one study declared any power analysis. We intended to assess publication bias, but the small number of studies for each outcome of interest were too few to derive any meaning from.

**Table 3:** The categories of risk of bias randomized clinical trials

Study	Randomization described	Allocation concealment reported	Intent to treat analysis performed	Blinded assessment stated	A priori power calculation performed	Total points	Risk of bias
Monini [9]	1	0	0.5	0.5	1	3	Low
Mezomo [3]	1	1	0	0.5	0	2.5	Moderate
Cohort studies							
Study	Representative sample of adequate size	Well matched sample	Adjusting for confounders	Blinded assessment stated	Reporting drop-outs	Total points	Risk of bias
Burrow [5]	1	1	0.5	0	0	2.5	Moderate
de Almeida [8]	1	1	0.5	0	0	2.5	Moderate
Machibya [7]	1	1	0.5	0	0	2.5	Moderate
Oz 2012 [4]	0.5	1	0.5	0	0	2	Moderate

Quality assessment: 1, criterion met; 0.5, criterion partially met; 0, criterion not met or not stated

Risk of bias: low, >4 points; moderate, 2-3.5 points; high, <2 points

### Description of outcomes

The studies were further divided into 2 categories based on the aspects of self-ligating brackets that were investigated: canine retraction rate and the amount of incisor and molar anchorage loss.

**Fig. 2:**
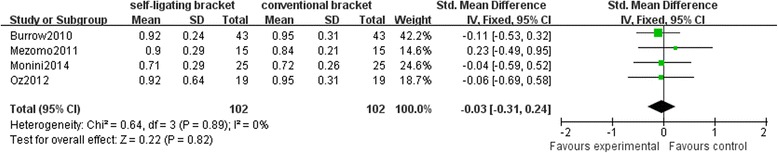
Canine retraction velocity

Four studies [3–5, 9] investigating the efficiency of SLBs compared with CBs were identified. Burrow et al. [5] compare the rates of retraction down an arch-wire of maxillary canine teeth when bracketed with a SLB was used on one side and a CB on the other. The rate of movement for the CB side was faster than that for other of the SLBs, with the Smart-Clip bracket faster than the Damon3 bracket. Although the mean differences at successive appointments were small, the difference between the CBs and the SLBs was statistically significant on a paired *t*-test. Mezomo et al. [3] found that there was no difference between SLB and CBs regarding the distal movement of upper canines. Monini et al. [9] evaluate the rate of canine retraction, anchorage loss and changes on canine and first molar inclinations using SLBs and CBs. Both brackets showed the same rate of canine retraction. Oz et al. [4] suggested that the rate of canine destabilization was not different between the two groups. Figure 2 shows the results of the meta-analysis from 4 eligible studies. No statistically significant difference was observed between the 2 groups in this outcome category.

**Fig. 3:**
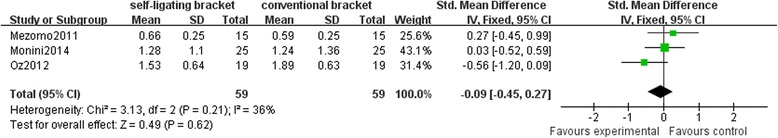
Molar mesial movement

**Fig. 4:**
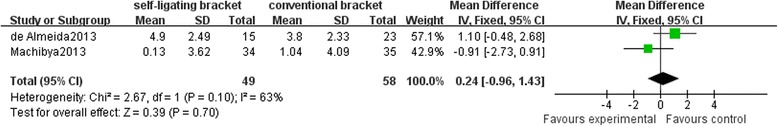
Changes of maxillary central incisor

**Fig. 5:**
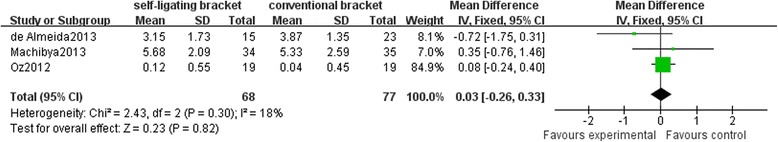
Changes of maxillary permanent molar

**Fig. 6:**
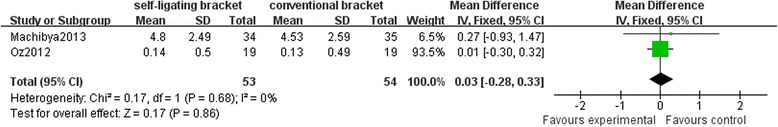
Changes of mandibular permanent molar

Five studies [3, 4, 7–9] investigating the incisor and molar anchorage loss of SLBs compared with CBs were identified. The outcomes studied maxillary central incisor (U1-Y) (mm), maxillary permanent molar (U6-Y) (mm), mandibular permanent molar (L6-Y)(mm), and molar mesial movement. Figure 3, 4, 5 and 6 shows the results of the meta-analysis from 5 eligible studies. No statistically significant difference was observed between the 2 groups in any outcome category.

## Discussion

There are many systematic reviews that help to identify and review the orthodontic literature with regards to the efficiency, effectiveness, and stability of treatments with SLBs compared with CBs [1, 10]. To the best of our knowledge, this systematic review was the first ever to be performed to provide data on the rate of canine retraction, and anchorage loss using SLBs and CBs.

Narrowing the inclusion criteria of studies increases homogeneity but also excludes the results of more trials and thus risks the exclusion of significant data [11]. This issue is important because the meta-analyses are frequently conducted on a limited number of RCTs. In these meta-analyses, more numbers from observational studies may aid in clinical information and establish a more solid foundation for causal inferences [11]. However, the potential biases are likely to be greater for non-randomized studies compared with RCTs, so results should be interpreted rigorously when they are included in reviews and meta-analyses [12]. The search strategy here only yielded 2 randomized studies on the adopted research. Thus, the results must be interpreted carefully.

The fact that some of these studies reviewed here have a different follow-up is a confounding factor, which varies from 3 months to space closure, to a completely closed space. A longer follow-up period lead to an increase in rate of anchorage loss, because other factors can influence molar anchorage loss from that point onward. This might have led to an underestimation of actual molar anchorage loss or tooth movement.

Another confounding factor is the fact that the studies including adolescents and adults patients. As we all know, the tooth movement was different and faster in adolescents than adults. To control as much as possible for the effect of age, future study should include subjects of comparable ages to those under orthodontic treatment. Moreover, the outcome measures were obtained using cephalometrics which may be result in some measure bias due to known intrinsic limitations of cephalometrics such as distortion and magnification. In some cases, the magnitude of error may approach the therapeutic changes and raise doubt about their validity [13]. Thus, in future study assess possible differences in canine retraction velocity and the amount of AP anchorage loss during maxillary canine retraction, applied three-dimensional measurement.

### Quality of the studies in this review

In this systematic review, 2 RCTs and 4 controlled clinical trials satisfied our inclusion criteria and were analyzed after searching and assessing the quality and the data extraction methods. We identified only 1 pertinent study with low risk of bias, 5 with moderate risk of bias. Therefore, the quality of most of the evidence in the meta-analyses is moderate to good. However, a prior sample size calculations was reported in only one studies, increasing the risk of false negative outcomes. The method of randomization and allocation concealment was often inadequate or incompletely reported. Intention-to-treat analysis would be a more appropriate technique ensuring consideration of all subjects initially randomized, maintaining the benefits of randomization throughout the trial. A CONSORT flow diagram is suggested as an appropriate way to improve the quality of data reported from parallel-group randomized trials, but just 2 studies used this method. Therefore, more prospective research in this area should be conducted and reported in accordance with the CONSORT guidelines, so that more high-quality RCTs will be eligible for future meta-analyses.

### Canine movement

Meta-analysis of the influence of bracket type on canine retraction rate confirmed that SLBs do not have a clinically significant improving canine movement. The four studies included in the meta-analysis had discordant findings; one [5] favored conventional brackets and the other three studies [3, 4, 9] demonstrated no difference between appliance systems. Clearly, tooth movement is influenced by a variety of factors, individual variations in biological response and tissue reactions to orthodontic movement may be critical.

Additionally, optimal orthodontic force produces excellent biological response with minimal tissue damage, resulting in rapid tooth movement with little discomfort, avoiding or minimizing hyalinized areas [14]. However, the magnitude and duration of the ideal force remains controversial [15]. The range force from 150 to 200 g employed in the including study may contribute to this discordant finding. Consequently, to definitively address these questions, a well-designed, prospective study of a large sample and similar force are required.

### Molar anchorage

Anchorage conservation is among the above advantages of SLB over CBs [16, 17]. Friction reduction during sliding mechanics is supposed to reduce the force needed to move teeth during orthodontic treatment, which in turn lowers the reciprocal force on anchor tooth or unit. This phenomenon is expected to improve anchorage and favor physiologic tooth movement, which may produce more stable treatment outcomes.

Five studies [3, 4, 7–9] investigating molar anchorage loss were identified. The meta-analysis showed no significant differences in the molar anchorage loss between the 2 groups, however, the anchorage loss ratio were various. In a randomized clinical trial, Mezomo et al. [3] found 2.68 mm of anchorage loss during the first 3 months of canine retraction for SLBs, while anchorage loss for CBs was 2.53 mm. On the other hand, Machibya et al. [7] found a mean 5.68 mm anchorage loss in the upper permanent molar for the SLB group compared to a mean loss of 5.33 mm in the CB group using en masse retraction for space closure. Monini [9] found a retraction to loss-of anchorage ratio of 5.4:1 for the SLB and 5.6:1 for the CB. The other two studies were found to have similar results [4, 8].

It is not surprising that no difference in molar anchorage loss was found between both bracket types. Overall, the type of ligation should have little influence over friction in space closure. Space closure may be more dependent on the second order wire-to bracket interface than on the first order, where design differences between brackets are apparent. Furthermore, other characteristics, such as bracket width, wire dimension, saliva, and occlusal forces, may be more important in affecting the frictional forces developed during canine retraction. This is supported by Thorensten and Kusy [18] who found that binding is not affected by the bracket ligation method once active configuration was reached.

### Limitation

The results of the present study has to be interpreted with caution because of its limitations. First of all, all confounding factors may have affected the canine retraction rate and the amount of AP anchorage loss. The lack of control of the confounding factors limited our potential to draw robust conclusions. Secondly, the number of randomized controlled trials included in the meta-analysis were limited, and individual studies had small sample sizes, and potential biases are likely to be greater for non-randomized studies compared with RCTs. Third, five databases were searched through and although every effort was made to identify all relevant studies, including studies in languages other than in English. Despite our criteria, it was unlikely that these databases would cover all the published, unpublished and ongoing studies relevant to our review. This therefore may have lead to a searching bias.

## Conclusion

The results of the present systematic review should be interpreted with caution due to the presence of uncontrolled, interpreted factors in the included studies. Within the limitations of the existing investigation the present study suggests that both brackets showed the same rate of canine retraction and loss of AP anchorage of the molars.

## Abbreviations

TAD: Temporary anchorage device
SLBs: Self-ligating brackets
AP: Antero-posterior
